# Exploring the Continuous Glucose Monitoring in Pediatric Diabetes: Current Practices, Innovative Metrics, and Future Implications

**DOI:** 10.3390/children11080907

**Published:** 2024-07-27

**Authors:** Agata Chobot, Claudia Piona, Bruno Bombaci, Olga Kamińska-Jackowiak, Valentina Mancioppi, Stefano Passanisi

**Affiliations:** 1 Department of Pediatrics, Institute of Medical Sciences, University of Opole, 45-040 Opole, Poland; agata.chobot@uni.opole.pl (A.C.); olga.kaminska-jackowiak@uni.opole.pl (O.K.-J.); 2 Department of Pediatrics, University Clinical Hospital in Opole, 46-020 Opole, Poland; 3 Pediatric Diabetes and Metabolic Disorders Unit, Regional Center for Pediatric Diabetes, University City Hospital, 37126 Verona, Italy; valentina.mancioppi@univr.it; 4 Department of Human Pathology in Adult and Developmental Age “Gaetano Barresi”, University of Messina, 98122 Messina, Italy; bruno.bombaci@studenti.unime.it (B.B.); stefano.passanisi@unime.it (S.P.)

**Keywords:** continuous glucose monitoring, CGM, pediatrics, children, time in range, time in tight range, diabetes, type 1 diabetes, type 2 diabetes

## Abstract

Continuous glucose monitoring (CGM) systems, including real-time CGM and intermittently scanned CGM, have revolutionized diabetes management, particularly in children and adolescents with type 1 diabetes (T1D). These systems provide detailed insights into glucose variability and detect asymptomatic and nocturnal hypoglycemia, addressing limitations of traditional self-monitoring blood glucose methods. CGM devices measure interstitial glucose concentrations constantly, enabling proactive therapeutic decisions and optimization of glycemic control through stored data analysis. CGM metrics such as time in range, time below range, and coefficient of variation are crucial for managing T1D, with emerging metrics like time in tight range and glycemia risk index showing potential for enhanced glycemic assessment. Recent advancements suggest the utility of CGM systems in monitoring the early stages of T1D and individuals with obesity complicated by pre-diabetes, highlighting its therapeutic versatility. This review discusses the current CGM systems for T1D during the pediatric age, established and emerging metrics, and future applications, emphasizing the critical role of CGM devices in improving glycemic control and clinical outcomes in children and adolescents with diabetes.

## 1. Introduction

The regular self-monitoring of glycemia is a pivotal element in diabetes management. The self-monitoring of blood glucose can be performed using self-monitoring capillary blood glucose (SMBG) methods and continuous glucose monitoring (CGM) systems, which include real-time CGM (RT-CGM) and intermittently scanned CGM (IS-CGM). In the SMBG method, although the actual number and regularity of fingerstick blood glucose measurements should be individualized, blood glucose measurements should usually be taken 6–10 times a day to monitor blood glucose adequately. It provides snapshots regarding glucose levels during the day but lacks data about glucose variability and asymptomatic and nocturnal hypoglycemia. CGM systems address these limitations, and their improved accuracy in recent years has increased their application safety [1]. CGM devices measure interstitial glucose concentrations every 1–15 min using enzyme-coated electrodes or fluorescence technology [1]. Constant access to the data about glucose fluctuations allows people with diabetes (PWDs) to make ongoing therapeutic decisions; stored and retrieved data may be analyzed by healthcare professionals during follow-ups, empowering the optimization of glycemic control. According to the current guidelines, CGM systems should be implemented to monitor glycemia in children and adolescents with type 1 diabetes (T1D) as soon as possible after diagnosis due to an impact on decreasing glycated hemoglobin (HbA1c) in the subsequent years of disease, and consequently on the risk of micro- and macrovascular complications in the future [1,2]. In adults, the use of continuous glucose monitoring systems is recommended mainly in people with T1D manifesting frequent episodes of hypoglycemia, especially with unawareness of hypoglycemia and during pregnancy in PWDs. In some countries, intensive insulin treatment has been suggested for type 2 and other types of diabetes in adults. Although glycated hemoglobin measurements remain the gold standard, CGM metrics should complement A1C in assessing glycemic control [3]. This concerns the most popular metrics such as time in range (TIR), the percentage of time that a person has glucose levels within the established range, time below range (TBR), the measure of time spent in hypoglycemia, and glucose variability assessed by coefficient of variation (CV), which is the standard deviation divided by arithmetic mean glucose. There is an ongoing discussion concerning other and novel CGM metrics—time in tight range (TITR) and glycemia risk index (GRI) [4,5]—and their significance in managing T1D [6,7,8].

Apart from the use of CGM in PWDs requiring insulin treatment, there are possible alternative applications. Dovc et al., in an article from 2024, summarize relevant research describing alternative implementations of CGM systems, among others: in stage 2 of T1D as indicators of progression to stage 3; in patients with type 2 diabetes (T2D) in non-insulin therapy; and people without diabetes [9]. The above research indicates the use of CGM systems not only as a tool for glycemic control and improvement in treatment outcomes in insulin-treated diabetes but also as a diagnostic tool for T1D staging or assessing the impact of novel drugs on improving glycemic control.

It should be noted that there is some diversity in the use of CGM devices in adults. These result, among others, are from the different body sizes, different studies performed in these populations, different registrations, and higher uptake of CGM systems integrated with insulin pumps (automated insulin delivery systems), which are recommended for treating T1D in children and adolescents.

This paper summarizes the present CGM systems registered for children with T1D and discusses the currently applicable CGM metrics and clinical targets, as well as presents new emerging CGM metrics and future implementation of CGM use in the pediatric age.

## 2. Clinical Practice of CGM in Diabetes in Children and Adolescents with T1D

### 2.1. CGM Devices Approved for the Pediatric Age

The application of CGM systems is a relevant factor leading to improvement in glycemic control [3,9,10]. In clinical use, there are two types of CGM devices: real-time CGM (RT-CGM) and intermittently scanned/flash CGM (is-CGM), as shown in Table 1 [11,12,13,14]. RT-CGM devices are registered for children with T1D aged 2 years old or older (Dexcom™, Northridge, CA, USA) [12] and 7 years old or older (Medtronic™ Guardian, Northridge, CA, USA) [13]. Depending on age, these transdermal sensors are self-inserted for 6–14 days in the back of the arm, abdomen, or upper buttocks. In addition, the Everense system is available for adults over 18 years of age, allowing the sensor to be replaced every 180 days. It is a device implanted in the subcutaneous tissue with no part placed on the skin [15]. CGM devices scan the glucose concentrations in the interstitial fluid every 1–15 min, providing near real-time glucose data that can be transferred to a reader or directly to the smartphone [2]. After reaching the set glycemic thresholds, the predictive alert informs the individual about imminent hypo- or hyperglycemia [2]. Glycemic data are stored and retrieved in the cloud and can be used during follow-ups by healthcare professionals. Sensor accuracy is described by the mean absolutive relative difference (MARD) between CGM readings and reference BG values [2]. Most commercially available sensors reach 8–10% MARD [2]. Recent generations of sensors (Dexcom G6, Dexcom G7, Medtronic Guardian 4) are non-adjuvant, meaning they are factory-calibrated and sensor glucose results do not need to be verified by capillary SMBG. However, under some circumstances—hypoglycemia, rapidly changing glycemia, and symptoms noncoherent to the CGM indications—SMBG should be performed before taking action [2].

Transdermal is-CGM devices (Freestyle Libre 1 and Freestyle Libre 2) are registered for children aged 4 years old or older and are self-inserted for up to 14 days [14]. Is-CGM uses a similar methodology to RT-CGM, but it requires scanning the sensor by holding a reader or smartphone close to or over the sensor; after that, the retrospective continuous glycemic metrics appear on the screen of the reader/smartphone [2,3]. Like RT-CGM, glycemic data may be stored and retrieved on the webserver. The most recent generation (Freestyle Libre 3) is registered for non-adjunctive use and provides access to glycemic data in real-time without scanning and real-time alarms, similar to RT-CGM [2].

Importantly, education, including interpretation of CGM-derived data, proper insertion, and skincare, has a crucial role in the effective and long-term use of CGM devices [2,16].

### 2.2. Glycemic Targets

Setting glycemic targets that both healthcare professionals and PWDs can work toward is essential for improving diabetes management. Although glycated hemoglobin remains the gold standard, it has limitations, such as a lack of information about acute glycemic events or intra- and interday glycemic variations [3]. Moreover, conditions associated with altered hemoglobin turnover, such as anemia, chronic kidney disease, and pregnancy, may affect Hb1AC measurements. As CGM addresses these limitations, the effective use of CGM-derived data requires clean clinical targets [3].

In February 2017, the ATTD Congress set up 14 core metrics for assessing CGM data [11]. The standardized CGM metrics should be reported as ambulatory glucose profiles (AGP) [11]. In clinical practice, “time in ranges” as a metric of glycemic control enables more effective diabetes management than HbA1c alone [3]. This glucose parameter includes three key CGM measurements: readings and time per day within the target glucose range (TIR), time spent below the target glucose range (time below range TBR), and above the target glucose range (time above range, TAR) expressed as percentages. The main aim of diabetes management is to increase the TIR without increasing the risk of hypoglycemia. Achieving TIR > 70% reflects a Hb1Ac of 7%; an increase of 10% in TIR correlates positively to a 0.5% increase in HbA1C [3]. Recent studies show evidence of the impact of TIR on microvascular complications such as retinopathy and microalbuminuria [17,18]. According to ISPAD 2022 guidelines, the expected time spent in each glycemic range for children and adolescents with T1D is as follows:

- >70% TIR between 3.9 and 10 mmol/L (70–180 mg/dL);
- <4% TBR (low) < 3.9 mmol/L (70 mg/dL);
- <1% TBR (very low) < 3.0 mmol/L (54 mg/dL);
- <25% TAR (high) >10 mmol/L (180 mg/dL);
- <5% TAR (very high) >13.9 mmol/L (250 mg/dL);
- Coefficient of variation (%CV) target ≤ 36% [2].

Less stringent targets are advisable only in particular situations associated with patient-centered therapy, such as limited access to insulin analogs and diabetes technology and psychological burden [1].

In newly diagnosed T1D, achieving TIR >80% without increasing the risk of hypoglycemia may be recommended due to the preservation of beta-cell function, leading to lower glycemic variability and a lower risk of hypoglycemia. Achieving glycemic control at the disease’s start may help preserve pancreatic beta-cell function longer [17].

Importantly, glycemic variability (assessed both in SMBG and CGM methods) seems to have a substantial impact on glycemic outcomes. In a study conducted on 805 children with T1D, decreasing glycemic variability correlates positively with a reduction in time above range and time below range [19]. Moreover, studies have shown that an increase in glycemic variability may be a risk factor for short-term and long-term complications of T1D [11].

The positive impact on glycemic outcomes depends on the amount of time the CGM is used. Based on published data, 70% use of CGM over the most recent 14 days correlates with 3 months’ mean glucose, time in ranges, and hyperglycemia metrics [3]. Correlations are weaker for hypoglycemia and glycemic variability [3]; therefore, longer observation may be required in individuals with more variable metabolic control. Also, another study conducted in the pediatric population with T1D showed that, in some specific subcategories of patients, a 4-week instead of 2-week period of CGM data may more accurately correlate with some of the glycemic metrics [19].

## 3. New Emerging CGM Metrics

In recent years, advancements in therapeutic tools have enabled youths with T1D to spend increasing amounts of time within the euglycemic range. This progress has led to the introduction of new metrics that may complement or potentially replace conventional metrics for a more insightful assessment of glycemic control in both clinical practice and experimental studies.

### 3.1. Time in Tight Range (TITR)

The most intriguing metric recently introduced is TITR, defined as the percentage of time spent in the euglycemic range between 70 and 140 mg/dL (3.9–7.8 mmol/L). This novel glucose control indicator has garnered significant interest within the scientific community. However, there is currently a paucity of real-world data in the literature analyzing TITR, hindering the establishment of feasible and applicable recommended targets for clinical practice.

**Figure 1:**
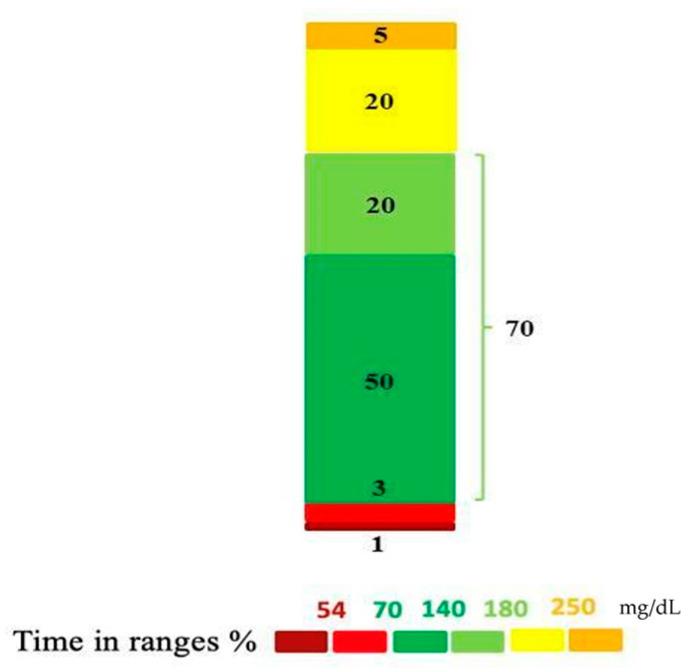
Graphical representation of the percentage of time spent in different glycemic ranges. Time in a tight range (TITR) corresponds to a darker green segment.

Preliminary data on this innovative metric from healthy individuals using CGM systems indicate that the median TITR in this population is approximately 96% [20]. The International Consensus Statement on CGM metrics for clinical trials [21] has proposed a desired threshold of 70%, similar to TIR, as a hypothetical target for clinical practice for individuals with T1D. More recently, this threshold has been adjusted to 50% based on real-world CGM analyses correlating TITR with other glucose control indicators (Figure 1). In a multicenter study involving 133 children and adolescents with T1D, Petersson et al. found that a TITR of 50% corresponded to an HbA1c value of approximately 6.5% [22]. Another real-world study on 854 pediatric subjects with T1D using various treatment modalities reported an overall mean TITR of 36.4% ± 12.8%. However, average TITR values (45% ± 11.2%) significantly increased when considering only study participants using automated insulin delivery systems [6]. The benefits of using advanced hybrid closed-loop devices in terms of TITR have also been demonstrated by Eviz et al., who reported that 87% of youths using these systems achieved the threshold of 50% [23].

The potential coexistence of TITR and TIR is widely debated. An Italian study revealed a strong linear correlation between these two metrics, with the threshold of TITR 50% corresponding to a TIR of 71.9% [6], closely aligning with the current recommended target of 70% [3]. An analysis of CGM data from nine studies involving 912 participants with T1D confirmed the strong correlation between TIR and TITR (r = 0.94). However, this relationship was reported to be non-linear and varied according to glycemic variability and time spent in hypoglycemia [8].

Assessing the relationship between TITR and glycemic variability emphasizes the added value of this novel metric in the clinical interpretation of CGM data. Lower glucose variability has been shown to significantly contribute to achieving desired TITR levels, suggesting that TITR may serve as an indicator of glycemic stability beyond being a mere advanced version of TIR [6,24].

A real-world study conducted by Dunn et al. involving 22,006 CGM users evaluated the relationship between mean glucose and the TIR/TITR ratio. The study found that TITR offers significant advantages over TIR for assessing glycemic status and progress toward stricter HbA1c goals, particularly as glucose levels approach normal ranges. The findings suggest that TITR may be more suitable than TIR for achieving HbA1c targets below 7%, whereas TIR tends to be less sensitive to changes in mean glucose levels and glycemic variability [24].

Another noteworthy aspect that emerged from a large cohort study is the lack of a correlation between TITR ≥ 50% and TBR [6]. This finding further supports the future application of TITR in routine clinical practice since the achievement of tighter glucose control does not appear to increase the risk of hypoglycemia.

Despite these encouraging data, several concerns have been raised about the potential psychological impact of the wide adoption of TITR, particularly regarding the anxiety associated with maintaining narrower glycemic control [25].

Finally, preliminary data suggest that implementing TITR in routine clinical practice for people with T1D is clinically relevant. They suggest that high TITR has a protective effect against the onset of long-term complications associated with T1D. Specifically, an analysis involving 808 adults with T1D showed an inverse association between TITR and microvascular complications [26].

### 3.2. Glycemia Risk Index (GRI)

**Figure 2:**
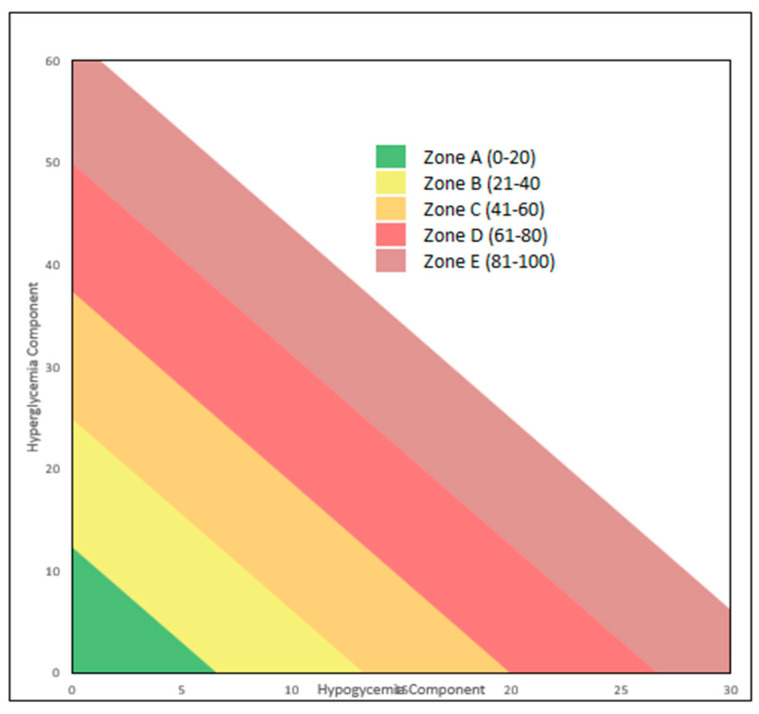
Graphical representation of glycemia risk index (GRI).

GRI has been proposed as a novel composite metric to evaluate the overall quality of glycemic control by capturing the risk associated with both hyperglycemia (calculated from TAR1 and TAR2) and hypoglycemia (calculated from TBR1 and TBR2) metrics [5]. The method for its computation was developed using the outcomes of a principal component analysis conducted on 2-week CGM profiles from 225 adults with diabetes who were on insulin therapy. This analysis identified two key components: one associated with hypoglycemia and the other with hyperglycemia [5]. The GRI can be visually depicted on a GRI Grid, with the hypoglycemia component plotted on the x-axis and the hyperglycemia component on the y-axis (Figure 2). Diagonal lines divide the grid into five zones (quintiles), representing the overall quality of glycemia from the best (0th to 20th percentile) to the worst (81st to 100th percentile) [5]. This graphical representation of the GRI allows clinicians to assess the hypoglycemia and hyperglycemia components for each subject over time. It also enables comparisons of glycemic risk among subjects based on their diabetes treatment strategies. Therefore, GRI could assist healthcare providers in assessing the effectiveness of diabetes management strategies and guiding treatment decisions to improve clinical outcomes for subjects with T1D [5].

Only a few studies have reported real-life data on GRI in adults and pediatric subjects with T1D.

Piona et al. were the first to calculate GRI from real-life CGM data of a large cohort of children and adolescents with T1D to assess its correlation with glycemic metrics and its usefulness in evaluating glycemic risk using different treatment strategies [4]. GRI positively correlated with hyperglycemia metrics, hypoglycemia metrics, mean glycemia, standard deviation, coefficient of variation, and HbA1c. The lowest value was found in subjects using Hybrid Closed Loop (HCL) systems, and the highest was found in subjects using multiple dose injection (MDI) combined with intermittently scanned CGM.

A multicenter, longitudinal, real-world study recruiting 368 children and adolescents with T1D who started using Minimed™ 780G with SmartGuard™ (Medtronic, Northridge, CA, USA) technology also demonstrated the effectiveness and safety of second-generation HCL systems in improving GRI after 1 year [26].

Panfil et al. retrospectively evaluated GRI utility for assessing glycemia quality between clinic visits in a cohort of 719 youth with T1D by analyzing its correlations with HbA1c measurements. Baseline GRI positively correlated with HbA1c at baseline and 3, 6, 9, and 12 months later [27].

Moreover, Morrison et al. evaluated the effects of exercise on GRI and CGM metrics within 24 h postexercise in 408 adults with T1D [28]. In this study, GRI significantly improved on exercise days compared to sedentary days. This change correlated to an increase in TBR and a reduction in TAR, both weighted in the GRI calculation.

In another study, Panfill et al. retrospectively assessed the relationship between GRI and T1D self-management habits in 1182 youth with T1D [29]. GRI decreased with the increasing number of habits performed, highlighting how youth engaged in more self-management habits achieved better glycemic control.

By correlating closely with clinical outcomes and expert evaluations of glycemic control, GRI is becoming an emerging metric indicating the overall quality of glucose management in subjects with T1D.

## 4. Future Implementation of CGM Use in the Pediatric Age

### 4.1. Prevention of T1D Complications

Poor glycemic control, traditionally measured by HbA1c, represents the major risk factor for diabetes complications. Several lines of evidence highlight the positive impact of CGM systems on glycemic outcomes, which recently led to the investigation of their possible impact on diabetes complications, including early signs of macrovascular complications and exposure to oxidative stress and cardiovascular risk factors (CVRFs) [30].

A cross-sectional study investigating risk factors for pre-clinical atherosclerosis in 267 children/adolescents with T1D showed no significant association between CGM metrics, oxidative stress, carotid intima-media thickness and carotid-femoral pulse wave velocity [31].

Piona et al. assessed the possible association between short-term glycemic control and variability measured by CGM and CVRFs in almost 900 children and adolescents with T1D. CGM metrics of glycemic control and variability, in particular TIR and CV, were significantly associated with the risk of being overweight in females and high LDL-c in both sexes [32].

Moreover, a systematic review by Yapanis et al. summarized the results of studies assessing the association between CGM metrics and microvascular and macrovascular complications in subjects with diabetes (both T1D and T2D), showing that higher TIR was associated with reduced risks of albuminuria, retinopathy, cardiovascular disease mortality, all-cause mortality, and abnormal carotid intima-media thickness [30].

This preliminary evidence supports the association between CVRF exposure, diabetes complications, and CGM-derived measures. In particular, TIR emerged as the most consistent measure. More longitudinal studies and trials are required to confirm these associations, as data for T1D are still limited.

### 4.2. Early Stages of T1D

The application of CGM systems has been explored in various clinical settings beyond established T1D. Notably, promising results have emerged from using these devices in individuals with early-stage T1D to identify those at high risk of progressing to stage 3 T1D.

A prospective study involving 91 children with positive T1D-specific autoantibodies showed that those who eventually developed overt diabetes had significantly higher average sensor glucose levels (119 vs. 105 mg/dL, *p* < 0.001) and greater glycemic variability. The children who progressed to diabetes spent 21% of their time with glucose levels above 140 mg/dL and 8% above 160 mg/dL, compared to 3% and 1%, respectively, for those who did not progress. The authors concluded that spending more than 10% of the time with glucose levels above 140 mg/dL is associated with a high risk of progression to overt diabetes within one year in children with positive antibodies [33].

Wilson et al. conducted a study to assess the risk of developing T1D in a cohort of 105 youths with stage 1 or stage 2 T1D or with negative T1D-specific autoantibodies. The researchers reported that progression to stage 3 disease was associated with the following CGM data: spending at least 5% of the time with glucose levels at or above 140 mg/dL (*p* = 0.01), at least 8% of the time with glucose levels at or above 140 mg/dL (*p* = 0.02), at least 5% of the time with glucose levels at or above 160 mg/dL (*p* < 0.001), and at least 8% of the time with glucose levels at or above 160 mg/dL [34].

A longitudinal study on 23 youths with positive T1D-specific autoantibodies found that those who progressed to diabetes during a median follow-up of 17.7 years had significantly increased baseline glycemic variability and mean glucose sensor levels. Spending ≥ 18% of the time with glucose levels above 140 mg/dL was identified as a predictor of progression to stage 3 T1D [35].

However, a study involving 93 subjects with multiple positive T1D-specific autoantibodies indicated that, while CGM use every 6 months can help predict the development of T1D, it is less effective than the traditional oral glucose tolerance test (OGTT). This suggests that there is currently insufficient evidence to replace OGTT measures with CGM in clinical trials [36].

### 4.3. CGM and Obesity

Obesity is one of the most severe and urgent public health issues, reaching epidemic proportions in both adult and pediatric populations in recent decades [37]. The obesity epidemic has led to an increase in the incidence and prevalence of T2D, with prediabetes already present in children and adolescents, with a prevalence of 13% in pediatric subjects with severe obesity [38]. According to the current ISPAD guidelines, prediabetes and T2D can be diagnosed based on a specific cut-off of fasting plasma glucose (FPG), glycated hemoglobin (HbA1c), and 2 h plasma glucose (2 h PG) during a glucose tolerance test (OGTT) [39]. As the development of prediabetes and its progression to T2D are continuous processes, the use of CGM in subjects with obesity offers the opportunity to evaluate glucose values in real life dynamically [39]. To date, data regarding the use of CGM as a diagnostic tool in subjects with obesity are limited, with initial evidence suggesting a potential role of glycemic variability (GV) metrics for the identification of subjects with early dysglycemia [40]. Indeed, average glucose exposure metrics in these subjects could not detect hyperglycemic spikes, which are typical of the postprandial phase and caused by defects in first-phase insulin secretion. On the contrary, GV metrics, in particular the coefficient of variation (CV), significantly increase from normoglycemic states (ranging from 13.4% to 22%) to pre-diabetes (15.3% to 28%) and are inversely related to insulin secretion indices [40]. Further studies are needed to confirm these data and more comprehensively evaluate the use of CGM and its metrics for the early diagnosis and treatment of prediabetes and T2D in subjects with obesity [40].

## 5. Conclusions

CGM systems address the limitations of the HbA1c measurements. They allow PWDs to make ongoing therapeutic decisions and strongly support optimizing glycemic control. However, CGM systems have some limitations that should be addressed. These include accuracy issues and variability in accuracy between different CGM systems; limited wear-time, requiring frequent sensor replacement; contact dermatitis (allergic and irritant) in the skin lesions sensor is attached; and sensor interference caused by substances and medications, which may lead to decreased sensor accuracy. Future research and technological development should focus on these limitations to improve CGM systems. Guidelines recommend implementing CGM systems to monitor glycemia in children and adolescents with T1D as soon as possible after diagnosis. Apart from currently applicable metrics and targets, new CGM metrics and targets, such as TITR and GRI, were shown to be clinically relevant. Future implementation of CGM in the pediatric population age may include early stages of T1D as well as obesity and T2D.

## Figures and Tables

**Table 1:** Continuous glucose monitoring (CGM) devices currently available for the pediatric population and their characteristics [12,13,14]. Eversense CGM system is registered for use in adults over 18.

	CGM Medtronic Guardian 4	CGM Medtronic Simplera	FreeStyle Libre 2	FreeStyle Libre 3	Dexcom G6	Dexcom G7	Dexcom One	Medtrum S9 CGM System
**Registration**	>7 years old	>2 years old	>4 years old	>4 years old	>2 years old	>2 years old	>2 years old	>2 years old
**Sensor lifespan**	7 days	7 days	14 days	14 days	10 days	10 days	10 days	14 days
**Glucose reading frequency**	Every 5 min	Every 5 min	Every 1 min	Every 1 min	Every 5 min	Every 5 min	Every 5 min	Every 2 min
**Wear location**	Back of the upper arm (7–17 years)	Back of upper arm Upper buttocks (2–17 years)	Back of the upper arm	Back of the upper arm	Back of the upper arm Abdomen Upper buttocks (2–17 years)	Back of the upper arm Upper buttocks (2–6 years)	Back of the upper arm Abdomen Upper buttocks (2–6 years)	Back of the upper arm Abdomen Upper buttocks (children)
**MARD**	10.6% (adults) 11.6% (children)	10.9% (children-arm) 10.2% (children-buttock)	9.2–9.5%	7.8–7.9%	9%	8.1% (arm)	8.7%	9.7%
**Alerts**	Yes	Yes	When scanned	Yes	Yes	Yes	Yes	Yes
**Warm-up time**	2 h	2 h	1 h	1 h	2 h	30 min	30 min	30 min

## Data Availability

Not applicable.
